# Leadless left ventricular endocardial pacing for CRT upgrades in previously failed and high-risk patients in comparison with coronary sinus CRT upgrades

**DOI:** 10.1093/europace/euab156

**Published:** 2021-07-28

**Authors:** Baldeep Singh Sidhu, Benjamin Sieniewicz, Justin Gould, Mark K Elliott, Vishal S Mehta, Timothy R Betts, Simon James, Andrew J Turley, Christian Butter, Martin Seifert, Lucas V A Boersma, Sam Riahi, Petr Neuzil, Mauro Biffi, Igor Diemberger, Pasquale Vergara, Martin Arnold, David T Keane, Pascal Defaye, Jean-Claude Deharo, Anthony Chow, Richard Schilling, Jonathan M Behar, Christophe Leclercq, Angelo Auricchio, Steven A Niederer, Christopher A Rinaldi

**Affiliations:** 1 School of Biomedical Engineering and Imaging Sciences, King's College London, UK; 2 Cardiology department, Guy's and St Thomas' NHS Foundation Trust, London, UK; 3 Oxford University Hospitals NHS Foundation Trust, Oxford, UK; 4 The James Cook Hospital, South Tees Hospitals NHS Foundation Trust, Middlesbrough, UK; 5 Immanuel Heart Center Bernau & Brandenburg Medical School Theodor Fontane, Germany; 6 St. Antonius Ziekenhuis, Nieuwegein, Utrecht, Netherlands/AUMC, Amsterdam, Netherlands; 7 Aalborg University Hospital, Aalborg, Denmark; 8 Na Homolce Hospital, Prague, Czech Republic; 9 IRCCS Policlinico S'Or 25 sola-Malpighi, Bologna, Italy; 10 San Raffaele Scientific Institute, Milan, Italy; 11 Friedrich-Alexander-Universität Erlangen-Nürnberg, Department of Cardiology, Erlangen, Germany; 12 St. Vincent's University Hospital, Dublin, Ireland; 13 CHU Grenoble Alpes, Grenoble, France; 14 Hopital La Timone, Marseille, France; 15 St. Bartholomew's Hospital, London, United Kingdom; 16 Univ Rennes, CHU Rennes, INSERM, LTSI-UMR 1099, F-35000 Rennes, France; 17 Fondazione Cardiocentro Ticino, Via Tesserete 48, Lugano, Switzerland

**Keywords:** Cardiac resynchronization therapy, Endocardial pacing, Epicardial pacing, WiSE-CRT system

## Abstract

**Aims:**

Cardiac resynchronization therapy (CRT) upgrades may be less likely to improve following intervention. Leadless left ventricular (LV) endocardial pacing has been used for patients with previously failed CRT or high-risk upgrades. We compared procedural and long-term outcomes in patients undergoing coronary sinus (CS) CRT upgrades with high-risk and previously failed CRT upgrades undergoing LV endocardial upgrades.

**Method and results:**

Prospective consecutive CS upgrades between 2015 and 2019 were compared with those undergoing WiSE-CRT implantation. Cardiac resynchronization therapy response at 6 months was defined as improvement in clinical composite score (CCS) and a reduction in LV end-systolic volume (LVESV) ≥15%. A total of 225 patients were analysed; 121 CS and 104 endocardial upgrades. Patients receiving WiSE-CRT tended to have more comorbidities and were more likely to have previous cardiac surgery (30.9% vs. 16.5%; *P *=* *0.012), hypertension (59.2% vs. 34.7%; *P *<* *0.001), chronic obstructive airways disease (19.4% vs. 9.9%; *P *=* *0.046), and chronic kidney disease (46.4% vs. 21.5%; *P *<* *0.01) but similar LV ejection fraction (30.0 ± 8.3% vs. 29.5 ± 8.6%; *P *=* *0.678). WiSE-CRT upgrades were successful in 97.1% with procedure-related mortality in 1.9%. Coronary sinus upgrades were successful in 97.5% of cases with a 2.5% rate of CS dissection and 5.6% lead malfunction/displacement. At 6 months, 91 WiSE-CRT upgrades and 107 CS upgrades had similar improvements in CCS (76.3% vs. 68.5%; *P *=* *0.210) and reduction in LVESV ≥15% (54.2% vs. 56.3%; *P *=* *0.835).

**Conclusion:**

Despite prior failed upgrades and high-risk patients with more comorbidities, WiSE-CRT upgrades had high rates of procedural success and similar improvements in CCS and LV remodelling with CS upgrades.

## Graphical Abstract

**Figure:**
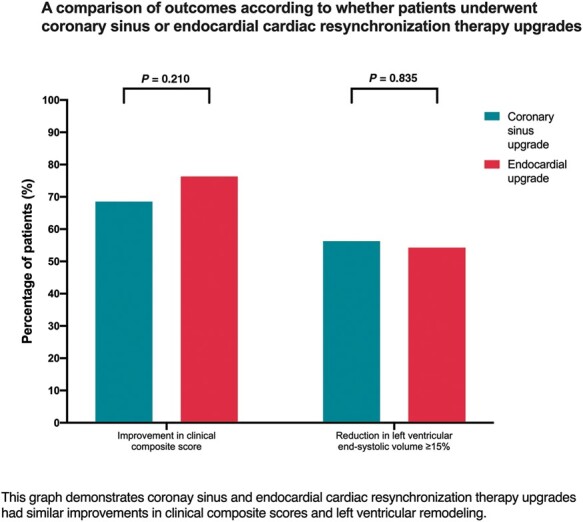

**What’s new?**

- Patients undergoing cardiac resynchronization therapy (CRT) upgrades have additional comorbidities that can increase the chance of non-response. Left ventricular (LV) endocardial pacing can be used in patients with previously failed CRT or high-risk upgrades.
- WiSE-CRT upgrades were successful in 97.1% of cases with procedure-related mortality in 1.9% and no acute strokes. There was one stroke during 6-month follow-up and one episode of sustained ventricular tachycardia.
- Coronary sinus (CS) upgrades were successful in 97.5% of cases with a 2.5% rate of CS dissection and 5.6% lead malfunction/displacement.
- Endocardial and CS upgrades demonstrated similar improvements in clinical composite score (76.3% vs. 68.5%; *P* = 0.210) and reduction in LV end-systolic volume ≥15% (54.2% vs. 56.3%; *P* = 0.835).
- These findings support the need for larger, randomized controlled trials with matched patient characteristics to determine whether endocardial pacing can lead to improved outcomes for CRT upgrades.

## Introduction

Cardiac resynchronization therapy (CRT) is an important intervention in symptomatic patients with severe left ventricular (LV) systolic impairment,^1^ with CRT upgrades accounting for 28% of all implantations.^2^ Patients undergoing CRT upgrades often suffer from additional comorbidities compared to *de novo* implantations and may respond differently.^3^^,^^4^ Conventional CRT upgrades with epicardial LV lead placement in the coronary sinus (CS) can be complicated by central venous occlusion that may require lead extraction or venoplasty to complete procedures, with additional associated risks.^5^^,^^6^ CS upgrade procedures can also be complicated by damage or displacement to previously implanted leads, leading to re-intervention with an increased risk of infection. Endocardial pacing may overcome many of these limitations allowing access to faster endocardial conduction and optimal pacing site selection avoiding myocardial scar and targeting areas of latest electrical or mechanical activation.^7^^,^^8^ Endocardial pacing has been shown to improve both left and right ventricular function.^9^ The WiSE-CRT system (EBR systems, USA) is capable of providing leadless LV endocardial pacing to achieve CRT. It consists of three separate components: a subcutaneous transmitter connected only to a subcutaneous battery and a leadless electrode within the left ventricle. Patients must have a co-implant *in situ* capable of providing continuous right ventricular pacing. Studies have demonstrated reliable biventricular pacing, resulting in improved patient symptoms and LV remodelling albeit with a risk of procedural-related major complications.^10–13^ Currently, eligibility for the WiSE-CRT system mandates patients meet additional inclusion criteria than for CS pacing that may result in a sicker patient cohort. We set out to compare procedural and long-term outcomes in patients undergoing CS CRT upgrades with patients with previously failed CRT or high-risk CRT upgrades receiving endocardial CRT upgrades with the WiSE-CRT system.

## Methods

### Study design

The study was approved by the Health Research Authority (20/HRA/0885). A prospective registry of patients undergoing both CS and endocardial CRT upgrades between 2015 and 2019 was collated. Patients were eligible to participate if they had a standard indication for CRT upgrade.^14^ In addition, patients undergoing WiSE-CRT implantation must either have been previously untreatable or considered a high-risk upgrade. Patients who were previously untreatable included those with a failed or unsuccessful CS lead placement or whose LV lead was subsequently programmed off due to a high threshold, no LV capture, phrenic nerve stimulation, lead displacement, or malfunction. High-risk upgrades included patients with a relative contraindication to CS lead implantation such as venous occlusion. Consecutive patients undergoing conventional CS CRT upgrades at Guy’s and St Thomas’ NHS Foundation Trust were compared with those who underwent CRT upgrade (excluding prior CRT non-responders) with the WiSE-CRT system in the WiCS-LV Post-Market Surveillance Registry (Clinical study number NCT02610673). A propensity score for the CS group was calculated by a logistic regression model using demographics that were significantly different at baseline. Endocardial upgrades were matched 1:1 with CS upgrades and this analysis is provided in the Supplementary material online.

### Coronary sinus cardiac resynchronization therapy upgrade procedure

Coronary sinus upgrades involved transvenous lead placement of a quadripolar LV lead in the lateral or posterolateral vein, wherever possible. Procedures were performed under sedation and local anaesthesia. Biventricular pacing was confirmed at the end of procedures, with devices programmed to simultaneous ventricular activation and an atrioventricular delay of 100–120 ms. The pacing vector that resulted in the narrowest QRS duration without phrenic nerve stimulation was chosen.

### WiSE-cardiac resynchronization therapy procedure

Patients with previously failed CRT or ‘high-risk’ CRT upgrades were included. Acoustic window screening was performed to identify suitable intercostal spaces that had no lung encroachment during maximal inspiration, with an angle between the ultrasound probe and basal LV posterolateral wall of <45°, distance <12 cm, and LV wall thickness ≥5 mm. Implantations were performed in a dual- or single-stage procedure with the latter involving implantation of the transmitter, battery, and receiver electrode during the same procedure. The transmitter and battery were initially implanted first to ensure the final electrode location could be tracked by the transmitter. Based on the optimal intercostal space identified during acoustic window screening, the transmitter was placed on the internal intercostal muscle and secured to the costal cartilage. Ultrasound screening intra-procedurally confirmed an adequate window and the battery was placed in the left mid-axillary line. The electrode was implanted using a retrograde aortic or transeptal approach to the LV endocardium. Intra-procedural testing to confirm right ventricular tracking and biventricular pacing was undertaken. The system only allows for simultaneous ventricular activation. Patients were discharged on dual anti-platelet therapy or if already on anticoagulation then an anti-platelet drug was added for 3 months post-intervention.

### Endpoints

Procedural-related major complications were collected, and patients were assessed at 6 months to determine their response to CRT on the basis of the clinical composite score (CCS) and LV remodelling as defined by transthoracic echocardiography. Patients were considered to have improved if (i) they had an improvement in their CCS consisting of alive, no heart failure hospitalizations, improvement in New York Heart Association (NYHA) functional class or global patient assessment^15^ and (ii) reduction in LV end-systolic volume (LVESV) ≥15%. Patients were considered to have worsened their CCS if they had died, experienced a heart failure hospitalization, worsening of NYHA functional class or global patient assessment and were considered to stabilize their CCS if they were alive, no heart failure hospitalization and no change in NYHA functional class or global patient assessment.

### Statistical analysis

Discrete data are presented as *n* values (with corresponding percentages) and continuous data as mean ± one standard deviation for normally distributed variables and median (interquartile range) for non-normally distributed variables. Discrete variables were compared using a *χ*^2^ or a Fisher’s exact test if the expected cell count was less than five. The Shapiro–Wilk test was used to assess the normality of continuous data, with a *P*-value ≥0.05 considered normally distributed. Normally distributed data were compared with an independent *t*-test and non-normally distributed data with a Wilcoxon signed-rank test. A two-sided *P*-value <0.05 was considered statistically significant. Statistical analyses were performed using Prism (GraphPad Software Inc., Version 9, CA, USA) and SPSS (IBM Switzerland, Version 26, Switzerland).

### Declaration of Helsinki

This study complies with the Declaration of Helsinki and locally appointed ethics committees have approved the research.

## Results

Overall, 225 patients undergoing CRT upgrade were included; 121 CS and 104 endocardial upgrades of which 65 were previously untreatable and 39 high-risk upgrades. In previously untreatable patients, 38 were due to failure of LV lead placement and 28 were due to the LV lead being programmed off. Baseline patient demographics are described in *Table 1*. Coronary sinus and endocardial upgrades were matched in terms of baseline demographics and comorbidities including age (70.9 ± 11.7 vs. 69.1 ± 10.2 years; *P *=* *0.110), male gender (80.2% vs. 78.9%; *P *=* *0.807), ischaemic aetiology (44.6% vs. 38.5%; *P *=* *0.350), atrial fibrillation (57.9% vs. 59.6%; *P *=* *0.789), diabetes mellitus (27.3 vs. 24.5; *P *=* *0.641), and LV ejection fraction (29.5 ± 8.6% vs. 30.0 ± 8.3%; *P *=* *0.678). Endocardial upgrades tended to have more comorbidities and were more likely to have a history of previous cardiac surgery (30.9% vs. 16.5%; *P *=* *0.012), hypertension (59.2% vs. 34.7%; *P *<* *0.001), chronic obstructive airways disease (19.4% vs. 9.9%; *P *=* *0.046), and chronic kidney disease (46.4% vs. 21.5%; *P *<* *0.001).

**Table 1:** Baseline patient demographics

Variable	Coronary sinus CRT upgrade	Endocardial CRT upgrade	*P*-value
Age, mean ± SD	70.9 ± 11.7	69.1 ± 10.2	0.110
Male, *n*/*N* (%)	97/121 (80.2)	82/104 (78.9)	0.807
Ischaemic cardiomyopathy, *n*/*N* (%)	54/121 (44.6)	40/104 (38.5)	0.350
Comorbidities, *n*/*N* (%)			
Cardiac surgery	20/121 (16.5)	30/97 (30.9)	0.012
Hypertension	42/121 (34.7)	58/98 (59.2)	<0.001
Atrial fibrillation	70/121 (57.9)	62/104 (59.6)	0.789
Diabetes mellitus	33/121 (27.3)	24/98 (24.5)	0.641
Chronic obstructive airways disease	12/121 (9.9)	19/98 (19.4)	0.046
Chronic kidney disease	26/121 (21.5)	45/97 (46.4)	<0.001
Cardiovascular accident	9/73 (12.33)	17/97 (17.5)	0.351
New York Heart Association functional class, mean ± SD	2.8 ± 0.7	2.6 ± 0.5	0.031
QRS duration, mean ± SD	170.6 ± 27.8	181.9 ± 30.2	0.008
Echocardiogram, mean ± SD			
Left ventricular ejection fraction	29.5 ± 8.6	30.0 ± 8.3	0.678
Left ventricular end-diastolic volume	186.2 ± 55.2	187.2 ± 90.3	0.246
Left ventricular end-systolic volume	129.8 ± 45.5	133.9 ± 75.7	0.499

CRT, cardiac resynchronization therapy; SD, standard deviation.

### Procedural outcomes

Coronary sinus CRT upgrades were successful in 118/121 (97.5%) patients. In three patients, the upgrade procedure was unsuccessful due to subclavian vein occlusion, CS stenosis preventing LV lead implantation, and failure of CS intubation due to an acute angle. There was evidence of subclavian vein stenosis at the entry site of the previous pacing leads in 14/121 (11.6%) patients who were overcome with either a medial puncture or a Terumo Glidewire^®^. In one patient, the right ventricular lead was damaged requiring an extra lead to be implanted. Additionally, 3/121 (2.5%) patients suffered a CS dissection, which were all managed conservatively without pericardial drainage. There were no acute procedure-related deaths, pericardial tamponades, or pneumothoraxes. One patient developed vegetations on their pacing leads at 5 months and died from sepsis despite system extraction. This patient did not undergo any other recent device interventions prior to the upgrade procedure and therefore their death is likely a complication of the upgrade procedure itself. Endocardial upgrades with the WiSE-CRT system were successful in 101/104 (97.1%) patients with biventricular pacing confirmed on a 12-lead electrocardiogram post-implantation. There was inconsistent LV capture in three patients; one due to lead placed in myocardial scar, one had a displaced transmitter requiring revision which improved capture, and one had inadequate capture despite transmitter revision. There were two (1.9%) procedure-related deaths; one death occurred suddenly 4 days post-procedure and a post-mortem revealed cardiac tamponade secondary to LV perforation and one patient presented to hospital 30 days post-procedure with septicaemia which was likely procedure-related. There were no acute cerebrovascular events.

### Six-month follow-up

#### Standard coronary sinus upgrades

**Figure 1:**
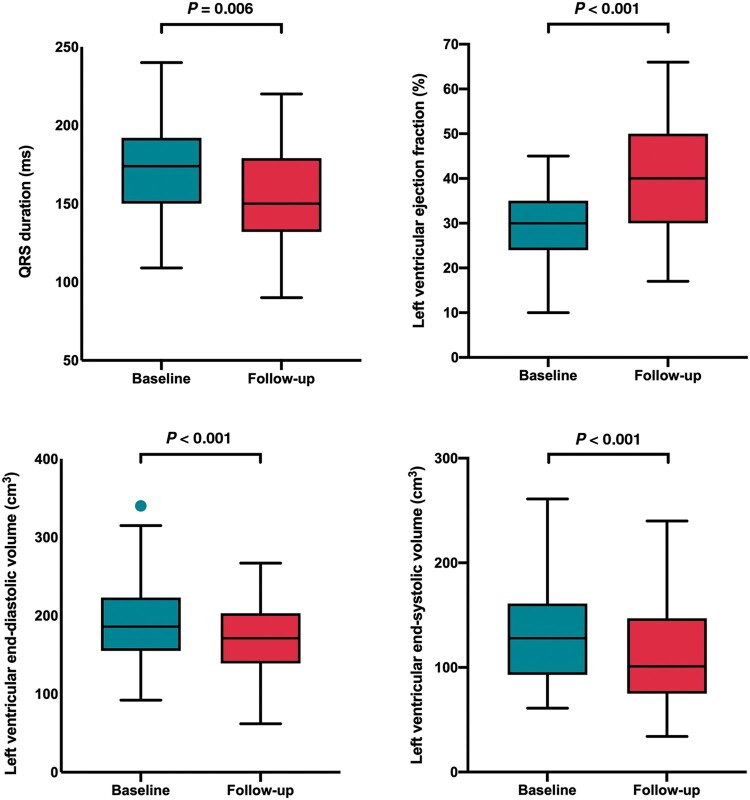
Box and whisker plots showing changes in QRS duration and left ventricular remodelling following coronary sinus cardiac resynchronization therapy upgrades.

One hundred and seven of 118 successfully implanted patients underwent assessment at 6-month follow-up. Four (3.3%) patients died within 6 months of implant, five were lost to follow-up, one had a displaced LV lead, and one had phrenic nerve stimulation in the only possible pacing vector. In the patients who died, three died from progressive heart failure and one patient suffered a device-related infection at 5 months requiring transvenous lead extraction and died from septicaemia. In addition, 6 (5.6%) patients suffered lead malfunction or displacement in the follow-up period (2 right atrial leads, 1 right ventricular lead, and 3 LV leads) with successful revision in 5 patients. At 6-month follow-up, there was a significant improvement in NYHA functional class (2.7 ± 0.7 vs. 1.9 ± 0.8; *P *<* *0.001), a reduction in QRS duration (171.5 ± 30.6 vs. 151.8 ± 30.0 ms; *P *=* *0.006), improvement in LV ejection fraction (30.0 ± 8.4% vs. 40.1 ± 12.1%; *P *<* *0.001), reduction in LV end-diastolic volume (186.8 ± 55.0 vs. 166.5 ± 52.4 cm^3^; *P *<* *0.001), and LVESV (129.1 ± 46.2 vs. 106.3 ± 49.3 cm^3^; *P *<* *0.001) (*Table 2 and Figure 1*). Overall, seven patients were admitted to hospital with decompensated heart failure, 68.5% showed an improvement in their CCS, 18.0% stabilized their CCS, 13.5% had worsening of their CCS, and 56.3% displayed a reduction in LVESV ≥15%.

**Figure 2:**
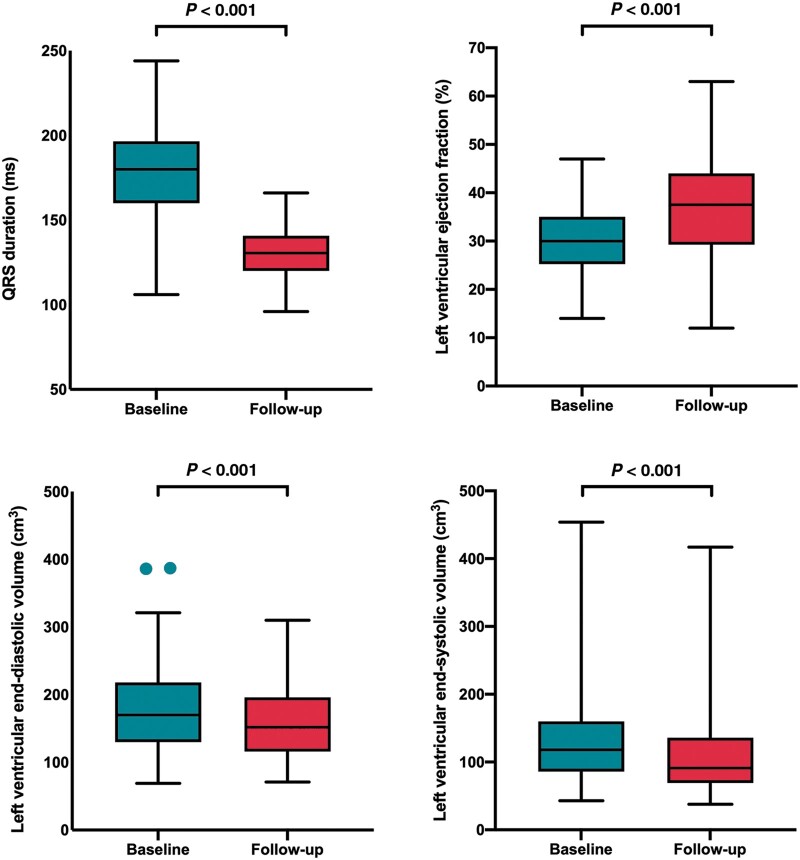
Box and whisker plots showing changes in QRS duration and left ventricular remodelling following endocardial cardiac resynchronization therapy upgrades with the WiSE-CRT system.

#### Endocardial upgrade follow-up

Ninety-one of 104 patients had 6-month follow-up. Three patients had inconsistent LV capture and four were lost to follow-up. Six (5.7%) patients died within 6 months: two suffered procedure-related deaths, one died at 3 months from incessant ventricular tachycardia, one died from an intracerebral haemorrhage at 2 months, 1 day after a ventricular tachycardia ablation whose arrhythmia pre-dated the WiSE-CRT implant, and two from non-cardiac related deaths. There were no other ventricular arrhythmias or ventricular high rates reported during the follow-up period and one patient suffered a stroke 5 months post-intervention. At 6-month follow-up, there was a significant reduction in NYHA functional class (2.6 ± 0.5 vs. 2.1 ± 0.6; *P *<* *0.001), reduction in QRS duration (181.1.5 ± 28.1 vs. 137.4 ± 28.2 ms; *P *<* *0.001), improvement in LV ejection fraction (30.8 ± 7.9% vs. 36.7 ± 10.4%; *P *<* *0.001), reduction in LV end-diastolic volume (187.1 ± 84.8 vs. 164.1 ± 75.3 cm^3^; *P *<* *0.001), and LVESV (131.7 ± 68.1 vs. 108.8 ± 65.3 cm^3^; *P *<* *0.001) (*Table 2* and *Figure 2*). Overall, two patients were admitted to hospital with decompensated heart failure, 76.3% showed an improvement in their CCS, 12.4% stabilized their CCS, 11.3% had worsening of their CCS, and 54.2% displayed a reduction in LVESV ≥15%. Sub-group analysis of patients who were previously untreatable or considered high-risk (*Table 2*) demonstrates a significant improvement in clinical response and LV remodelling with the WiSE-CRT system.

**Table 2:** Coronary sinus and endocardial upgrade outcomes

Variable	Baseline, mean ± SD	Follow-up, mean ± SD	*P*-value
Coronary sinus upgrade			
New York Heart Association functional class	2.7 ± 0.7	1.9 ± 0.8	<0.001
QRS duration	171.5 ± 30.6	151.8 ± 30.0	0.006
Echocardiogram			
Left ventricular ejection fraction	30.0 ± 8.4	40.1 ± 12.1	<0.001
Left ventricular end-diastolic volume	186.8 ± 55.0	166.5 ± 52.4	<0.001
Left ventricular end-systolic volume	129.1 ± 46.2	106.3 ± 49.3	<0.001
Endocardial upgrades overall			
New York Heart Association functional class	2.6 ± 0.5	2.1 ± 0.6	<0.001
QRS duration	181.1 ± 28.1	137.4 ± 28.2	<0.001
Echocardiogram			
Left ventricular ejection fraction	30.8 ± 7.9	36.7 ± 10.4	<0.001
Left ventricular end-diastolic volume	187.1 ± 84.8	164.1 ± 75.3	<0.001
Left ventricular end-systolic volume	131.7 ± 68.1	108.8 ± 65.3	<0.001
Previously untreatable			
New York Heart Association functional class	2.6 ± 0.5	2.1 ± 0.6	<0.001
QRS duration	180.4 ± 29.9	139.0 ± 30.2	<0.001
Echocardiogram			
Left ventricular ejection fraction	31.4 ± 8.2	36.2 ± 10.4	0.005
Left ventricular end-diastolic volume	192.4 ± 104.8	174.5 ± 89.5	0.071
Left ventricular end-systolic volume	136.6 ± 84.4	118.2 ± 75.9	0.010
High-risk upgrade			
New York Heart Association functional class	2.5 ± 0.6	2.1 ± 0.7	0.004
QRS duration	184.0 ± 16.4	128.9 ± 11.2	<0.001
Echocardiogram			
Left ventricular ejection fraction	30.0 ± 7.5	37.4 ± 10.4	<0.001
Left ventricular end-diastolic volume	180.4 ± 50.4	150.9 ± 50.8	<0.001
Left ventricular end-systolic volume	125.5 ± 39.7	96.9 ± 47.5	<0.001

SD, standard deviation

### Comparison of coronary sinus and endocardial upgrades

The outcomes of patients proceeding to follow-up were compared; 107 CS and 91 endocardial upgrades (*Table 3*). Patients with endocardial upgrades had a greater absolute reduction in QRS duration (−43.7 ± 31.6 vs. −19.7 ± 35.9 ms; *P *=* *0.002). They both had a similar absolute reduction in LV end-diastolic volume (23.0 ± 42.2 vs. 20.3 ± 37.6 cm^3^; *P *=* *0.736) and LVESV (−22.9 ± 35.5 vs. −22.7 ± 34.9 cm^3^; *P *=* *0.807) but less likely to have an absolute change in LV ejection fraction (5.9 ± 8.9% vs. 10.1 ± 10.2%; *P *=* *0.008). They had similar improvements in CCS (76.3% vs. 68.5%; *P *=* *0.210) and reduction in LVESV ≥15% (54.2% vs. 56.3%; *P *=* *0.835). Ischaemic patients undergoing endocardial and CS upgrades demonstrated similar improvements in CCS (79.0% vs. 66.0%; *P *=* *0.182) and reduction in LVESV ≥15% (58.3% vs. 36.4%; *P *=* *0.136). A propensity score showed patients undergoing endocardial and CS upgrades had similar improvements in CCS (72.7% vs. 70.8%; *P *=* *0.804) and reduction in LVESV ≥15% (51.3% vs. 63.6%; *P *=* *0.295) (Supplementary material online).

**Table 3:** Comparison of coronary sinus and endocardial CRT upgrades

Variable	Coronary sinus CRT upgrade, mean ± SD	Endocardial CRT upgrade, mean ± SD	*P*-value
New York Heart Association functional class			
Absolute change	−0.8 ± 0.8	−0.5 ± 0.7	<0.001
Percentage change	−28.7 ± 26.7	−16.4 ± 25.9	<0.001
QRS duration			
Absolute change	−19.7 ± 35.9	−43.7 ± 31.6	0.002
Percentage change	−9.7 ± 21.0	−23.1 ± 16.0	0.002
Left ventricular ejection fraction			
Absolute change	10.1 ± 10.2	5.9 ± 8.9	0.008
Left ventricular end-diastolic volume			
Absolute change	−20.3 ± 37.6	−23.0 ± 42.2	0.736
Percentage change	−8.9 ± 22.8	−9.9 ± 22.5	0.995
Left ventricular end-systolic volume			
Absolute change	−22.7 ± 34.9	−22.9 ± 35.5	0.807
Percentage change	−17.5 ± 32.3	−15.8 ± 28.6	0.652

CRT, cardiac resynchronization therapy; SD, standard deviation.

Sub-group analysis of patients previously untreatable who underwent endocardial upgrades compared with CS upgrades showed similar improvements in CCS (78.0% vs. 68.5%; *P *=* *0.192) and reduction in LVESV ≥15% (39.4% vs. 56.3%; *P *=* *0.138). Similarly, high-risk patients who underwent endocardial upgrades compared with CS upgrades showed similar improvement in CCS (73.7% vs. 68.5%; *P *=* *0.547) and reduction in LVESV ≥15% (73.1% vs. 56.3%; *P *=* *0.157).

## Discussion

We compared the outcomes of patients with previously failed or high-risk CRT upgrades undergoing WiSE-CRT implantation with patients undergoing standard CRT upgrades. The predominant findings were as follows:

1. Coronary sinus upgrades were successful in 97.5% of cases, with evidence of a significant venous stenosis in 11.6% and no procedure-related mortality but 4.6% required re-intervention for lead malfunction/displacement. Patients displayed significant improvement in clinical and echocardiographic outcomes following CRT.
2. Endocardial upgrades were performed in patients with more comorbidities and were successful in 97.1% of cases with 1.9% procedure-related mortality. There were no acute stroke and during the follow-up period, one patient suffered a stroke and one patient a ventricular arrhythmia. Patients displayed significant improvement in clinical and echocardiographic outcomes following intervention.
3. Patients undergoing CS and endocardial upgrades had similar improvements in their CCS and reduction in LVESV ≥15%. Coronary sinus upgrades were more likely to have an absolute change in LV ejection fraction.
4. There was a higher burden of comorbidities in the endocardial group, reflecting real-world CRT practice,^16^ but the propensity-matched score indicated these benefits persisted in this high-risk group.

### Comparison with previous studies

A direct comparison of CS and endocardial upgrades has not been previously investigated, to the best of our knowledge, but each intervention has been studied separately. Several trials have reported outcomes following CS upgrades.^2^^,^^3^^,^^17^ In a European CRT survey involving 692 upgrade procedures in 141 centres, they found 73% of patients improved their global assessment at 1 year.^2^ Significant procedure-related complications included 0.3% rate of cardiac tamponade, 1.7% CS dissection, and 3.2% experienced a lead displacement. A prospectively collected multicentre study of 177 upgrade procedures found 57% of patients improved their NYHA functional status and overall, there was a mean improvement in LV ejection fraction of 2.9 ± 9%.^18^ In the current study of 121 CS upgrade procedures, we found similar rates of CS dissection (2.5%) and lead malfunction/displacement (5.6%) but significantly better rates of clinical and echocardiographic response. Endocardial pacing using lead-based technology was investigated in a multicentre study of 138 patients.^19^ The inclusion criteria were similar to the present study and also included patients who were non-responders to conventional CRT. They found a success rate of 89.4%, with 6.8% experiencing transient ischaemic attacks and 3.8% non-disabling strokes. At follow-up, 59% improved their NYHA functional class and 55% had a reduction in LVESV ≥15%. The position of the LV lead could only be fixated to the desired location in 81% of implants, arguably the greatest potential benefit of endocardial pacing. The current study compares favourably with these results since we have shown higher success rates and comparable clinical and echocardiographic improvements.

### Clinical perspective

Currently, patients undergoing WiSE-CRT implantation have relatively few alternative options to achieve resynchronization, evidenced by the inclusion criteria for the device. These patients may represent a higher risk group with more comorbidities than those felt suitable for conventional CS upgrades. Supportively, in the current study endocardial upgrade patients were significantly more likely to have had previous cardiac surgery, hypertension, and chronic kidney disease. Despite these additional comorbidities these patients had similar improvements in CCS and reduction in LVESV ≥15% compared with CS pacing. Furthermore, 88.7% of endocardial upgrades had improvement or stabilization of their CCS which has important prognostic implications.^20^ Indeed, high-risk upgrades unlike previously untreatable patients have never received resynchronization therapy and in these patients, there was a non-significant trend towards improved clinical response and LV remodelling compared with standard upgrades. Additionally, previously untreatable patients who received the WiSE-CRT system represent the highest-risk patient cohort but still had similar outcomes to CS pacing. A potential benefit of the WiSE-CRT system is the ability to pace anywhere inside the LV and provide targeted pacing, with guidance shown to improve outcomes.^7^^,^^8^ The current study showed ischaemic patients undergoing upgrades had similar improvements in CCS and LV remodelling, although there was a non-significant trend favouring endocardial pacing. This study was not powered to detect a significant difference in outcomes of ischaemic patients, but endocardial pacing may potentially be more beneficial due to site-specific pacing. Randomized controlled trials of matched patients undergoing each intervention will be important to further investigate outcomes and determine whether ischaemic patients are more likely to benefit with endocardial pacing. In addition, conduction system pacing may be beneficial in patients who fail conventional CRT upgrades and left bundle branch area pacing may be possible with the WiSE-CRT system. Randomized controlled trials are needed to determine whether endocardial pacing with the WiSE-CRT system or conduction system pacing may prove to be more beneficial than conventional upgrades.

### Limitations

This study is subject to the same limitations as any prospectively collected data, although data collection was standardized to reduce bias. There is inclusion bias as patients undergoing WiSE-CRT implantation had either failed prior CRT upgrades or were felt to be high risk. We did not perform a matched analysis as this would have resulted in a smaller study size which may potentially have led to bias and inaccurate results. However, both groups were matched in terms of important baseline demographics including age, sex, aetiology, atrial fibrillation, LV ejection fraction, LV end-diastolic volume, and LVESV. Biventricular pacing was estimated at 6 months from device interrogation but cannot be fully relied upon without attaching a Holter monitor for QRS morphology. The procedural duration was not documented in all patients in both arms and this would have been important since the invasive nature of WiSE-CRT procedures may have prolonged interventions. Changes in optimal medical therapy or anti-arrhythmic treatment during the study were not recorded and this would have been important as it may have influenced LV remodelling. However, all patients must have been on optimal medical therapy prior to enrolment into the study and therefore we would not expect any significant medication changes during follow-up. The right ventricular pacing burden was not collected during the study and this would have been important to determine the relative proportion of pacing-induced cardiomyopathy.

## Conclusion

Patients currently indicated for WiSE-CRT upgrades are a complex patient cohort with multiple comorbidities and may be less likely to respond to CRT with prior failed upgrade attempts or high-risk features precluding standard upgrade. Despite this WiSE-CRT upgrades showed a high rate of procedural success with 1.9% rate of procedure-related mortality and similar rate of improvements in CCS and reduction in LVESV ≥15% compared to patients undergoing CS upgrades. These findings support the need for larger, randomized controlled trials with matched patient characteristics to determine whether endocardial pacing can lead to improved outcomes for CRT upgrades.

## Supplementary material

Supplementary material is available at *Europace* online.

## Supplementary Material

euab156_supplementary_data
