# Supplementary material for: Leadless left ventricular endocardial pacing for CRT upgrades in previously failed and high-risk patients in comparison with coronary sinus CRT upgrades
Source: Europace. 2021 Jul 28;23(10):1577–85. doi: 10.1093/europace/euab156 (PMC8502498; doi:10.1093/europace/euab156)
Supplement: euab156_supplementary_data [file euab156_supplementary_data.docx]

Supplementary file

The propensity score for the epicardial group was calculated by a logistic regression model using the demographics which were significantly different at baseline, and these included chronic kidney disease, chronic obstructive pulmonary disease and New York Heart Association functional class. Endocardial upgrade recipients were matched 1:1 to coronary sinus upgrade recipients by their propensity scores, using the nearest neighbour method with a calliper of 0.20 and no replacements (R-studio, package *Matchit*)^1^. An overall standardized mean difference of 0.0126 suggested balanced datasets^2^, as does the histogram demonstrating the propensity scores distributions of the groups (supplementary figure S1). Overall, 58 patients in each intervention proceed to analysis. Patients undergoing endocardial and coronary sinus upgrades had similar improvements in clinical composite score (72.7 vs 70.8%; *P* = 0.804) and reduction in left ventricular end-systolic volume ≥15% (51.3 vs. 63.6%; *P* = 0.295).


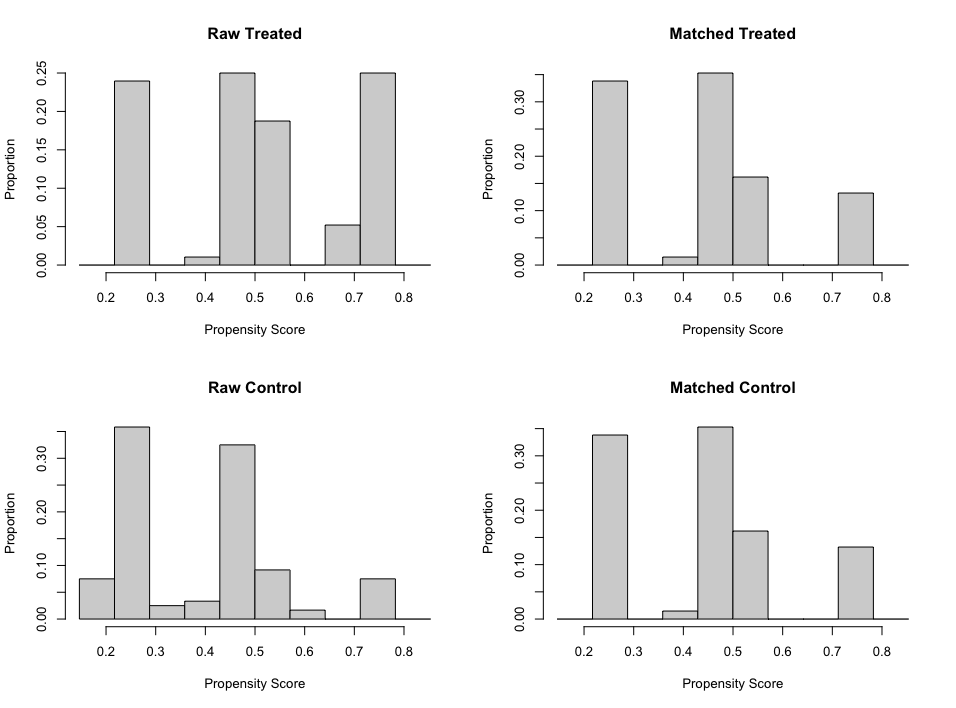


Supplementary Figure S1 - Histograms showing the density of propensity score distribution in the treated and control groups before and after matching

Supplementary References

1. Ho DE, King G, Stuart EA, Imai K: MatchIt. Nonparametric Preprocessing for. J Stat Softw [Internet] 2011; 42:1–28. Available from: http://www.jstatsoft.org/v42/i08/paper

2. Zhang Z, Kim HJ, Lonjon G, Zhu Y. Balance diagnostics after propensity score matching. Ann Transl Med 2019; 7:16–16.
